# Supplementary material for: Enhanced SARS-CoV-2-Specific CD4+ T Cell Activation and Multifunctionality in Late Convalescent COVID-19 Individuals
Source: Viruses. 2022 Mar 2;14(3):511. doi: 10.3390/v14030511 (PMC8954911; doi:10.3390/v14030511)
Supplement: Supplementary file 1 [file viruses-14-00511-s001.zip › viruses-1565345-supplementary.pdf]

Figure S1

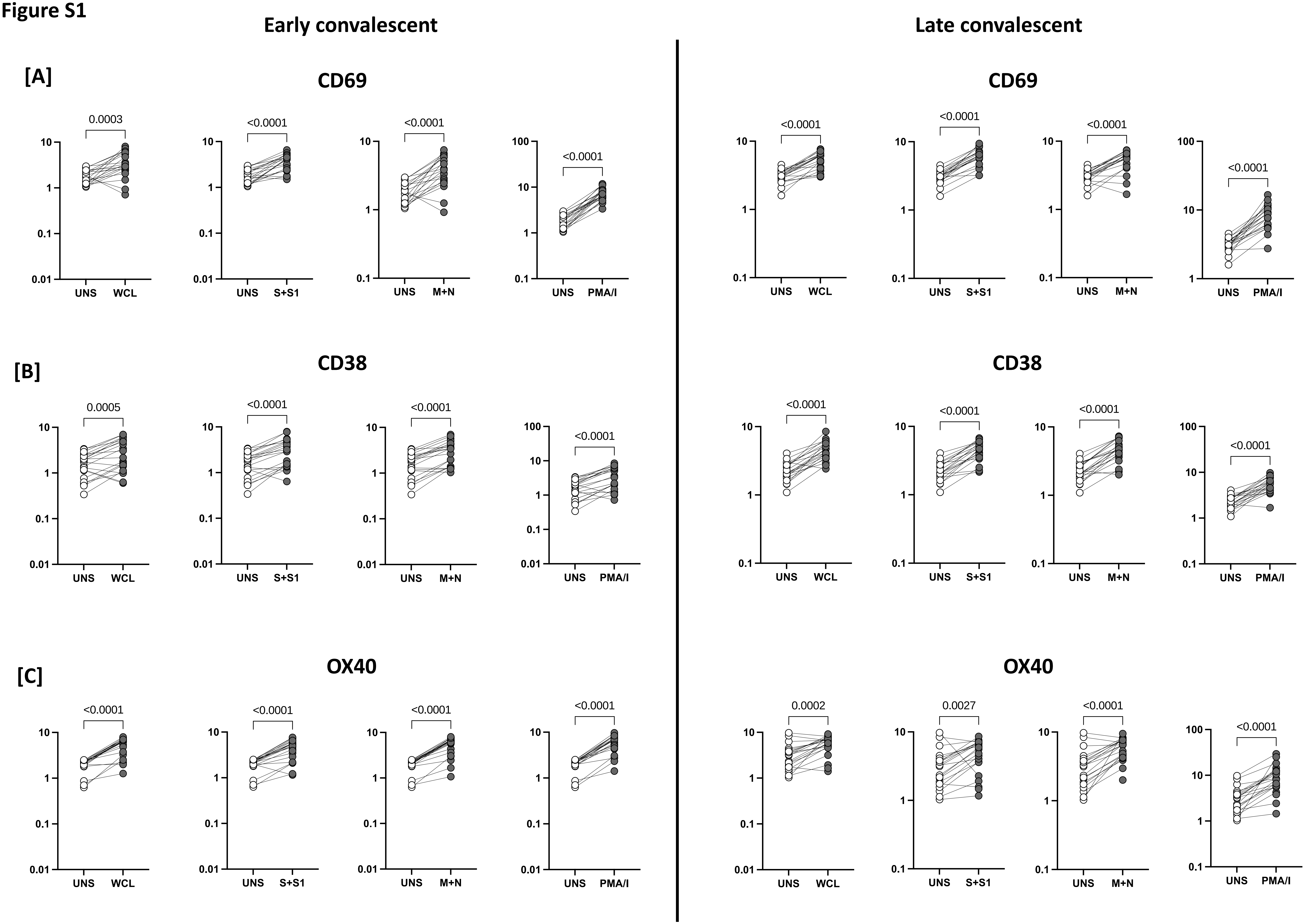

Figure S1: The frequencies of activation markers were estimated in ECV and LCV individuals upon no stimulation and SARS-CoV-2 antigen stimulation. Each circle represents a single individual and the bars represent the geometric mean values *P* values were calculated using the Wilcoxon matched pair test.

Figure S2

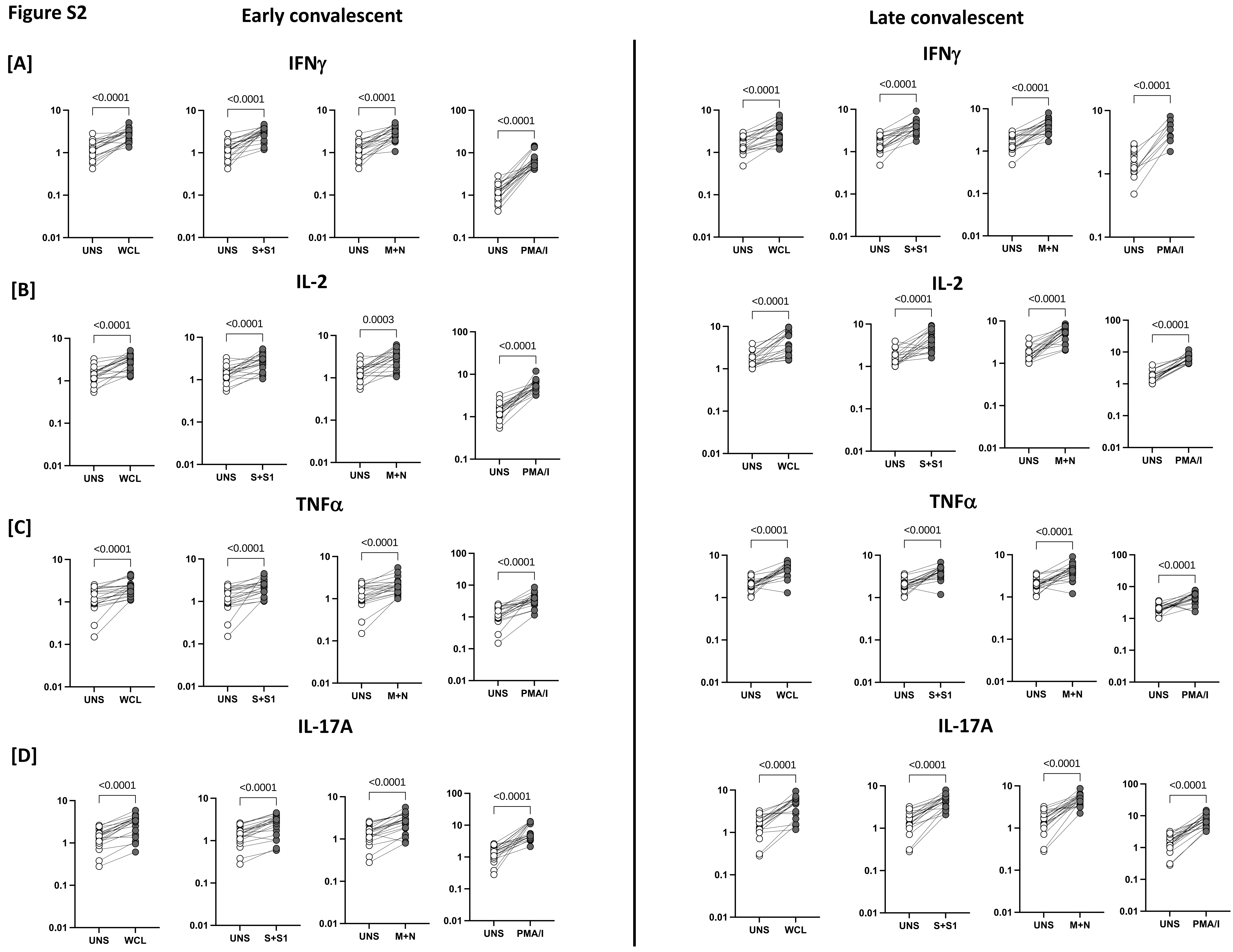

Early convalescent
Late convalescent
[A]
IFNγ
<0.0001
0.0003
UNS
WCL
S+S1
M+N
PMA/I
[B]
IL-2
[C]
TNFα
[D]
IL-17A
100
10
1
0.1
0.01

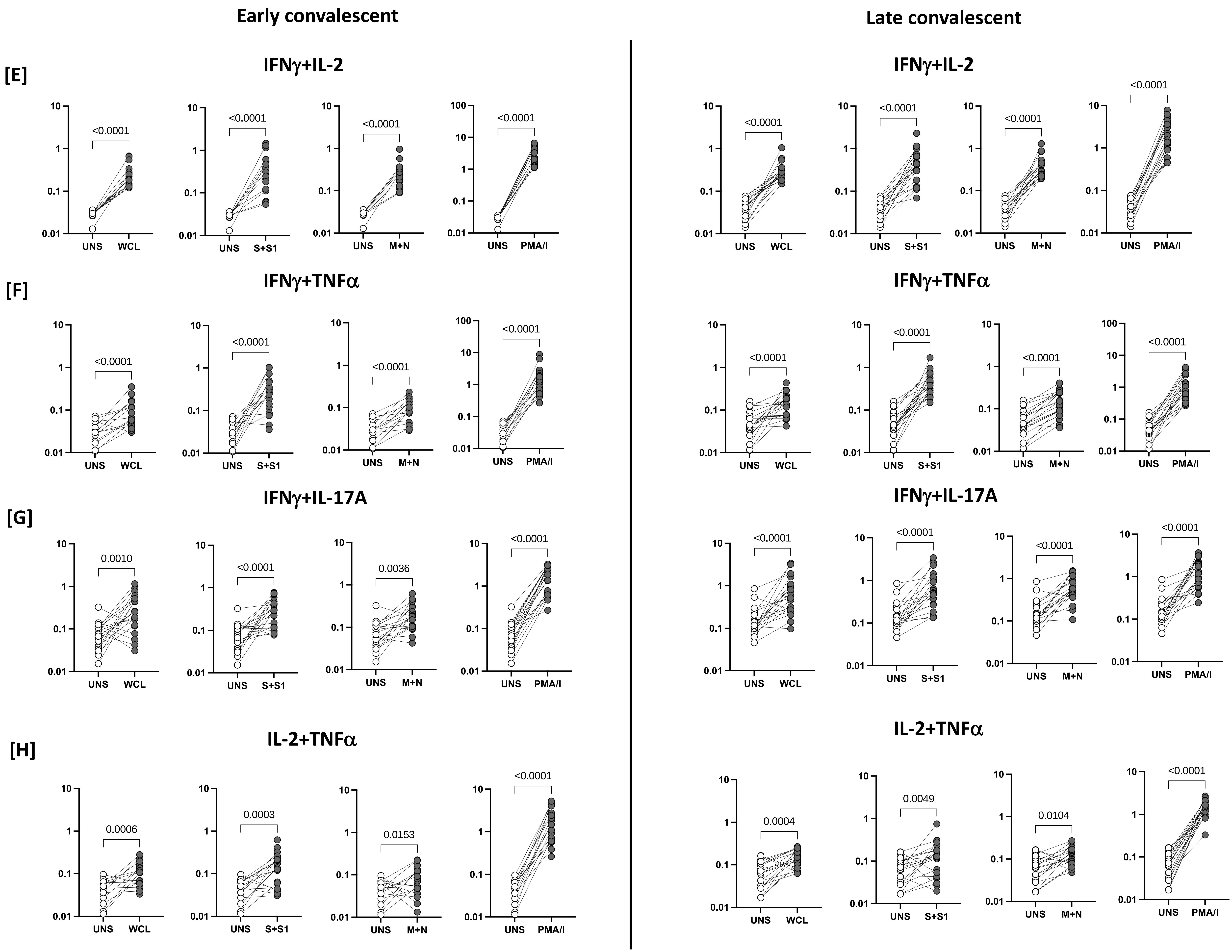
Early convalescent
Late convalescent
[E]
IFNγ+IL-2
<0.0001
<0.0001
<0.0001
<0.0001
<0.0001
<0.0001
<0.0001
<0.0001
[F]
IFNγ+TNFα
<0.0001
<0.0001
<0.0001
<0.0001
<0.0001
<0.0001
<0.0001
<0.0001
[G]
IFNγ+IL-17A
0.0010
<0.0001
0.0036
<0.0001
<0.0001
<0.0001
<0.0001
<0.0001
[H]
IL-2+TNFα
0.0006
0.0003
0.0153
<0.0001
0.0004
0.0049
0.0104
<0.0001
UNS
WCL
S+S1
M+N
PMA/I
0.01
0.1
1
10
100

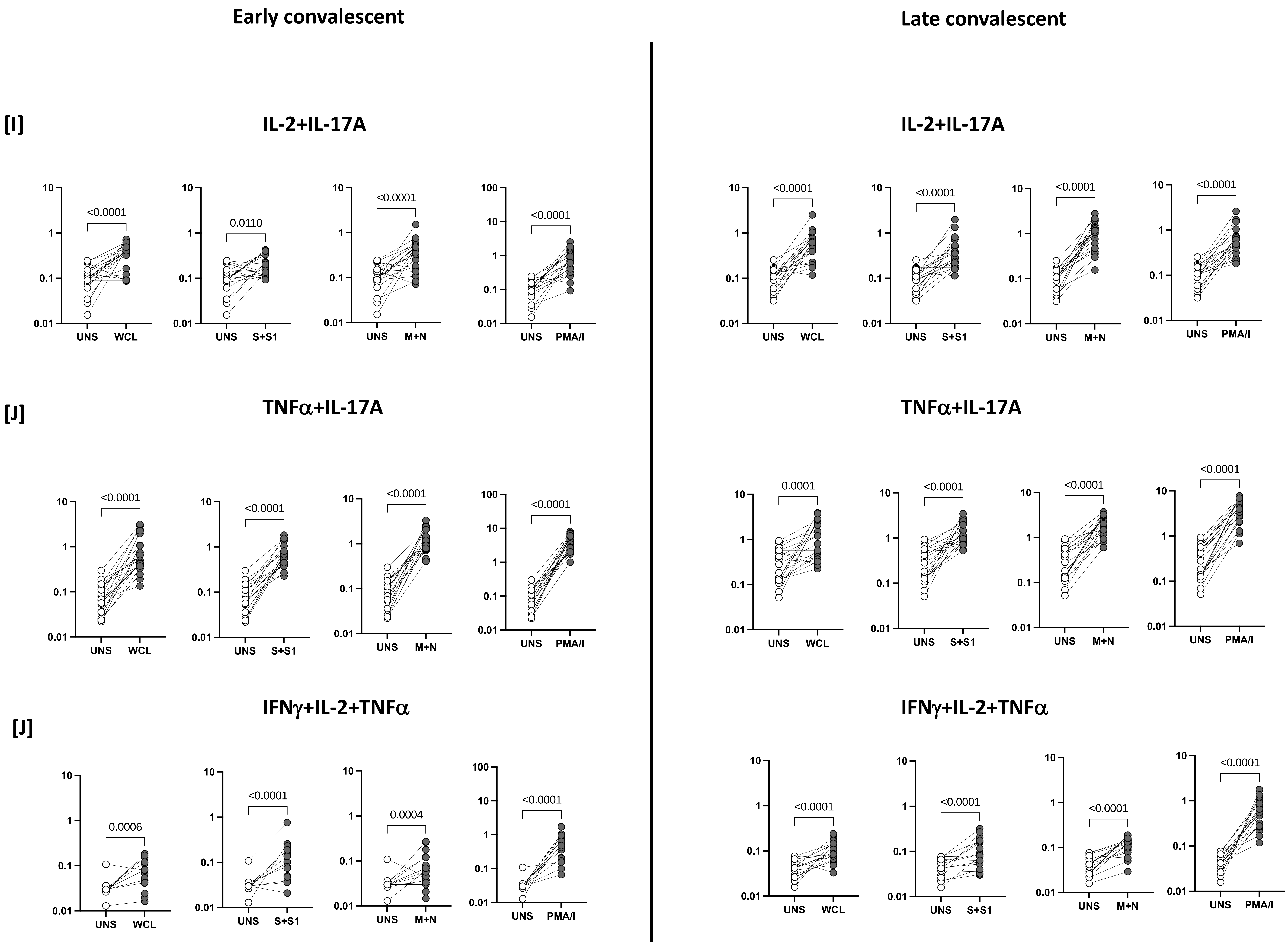

Figure S2: The frequencies of multifunctional cytokines were estimated in ECV and LCV individuals upon no stimulation and SARS-CoV-2 antigen stimulation. Each circle represents a single individual and the bars represent the geometric mean values *P* values were calculated using the Wilcoxon matched pair test.

Figure S3

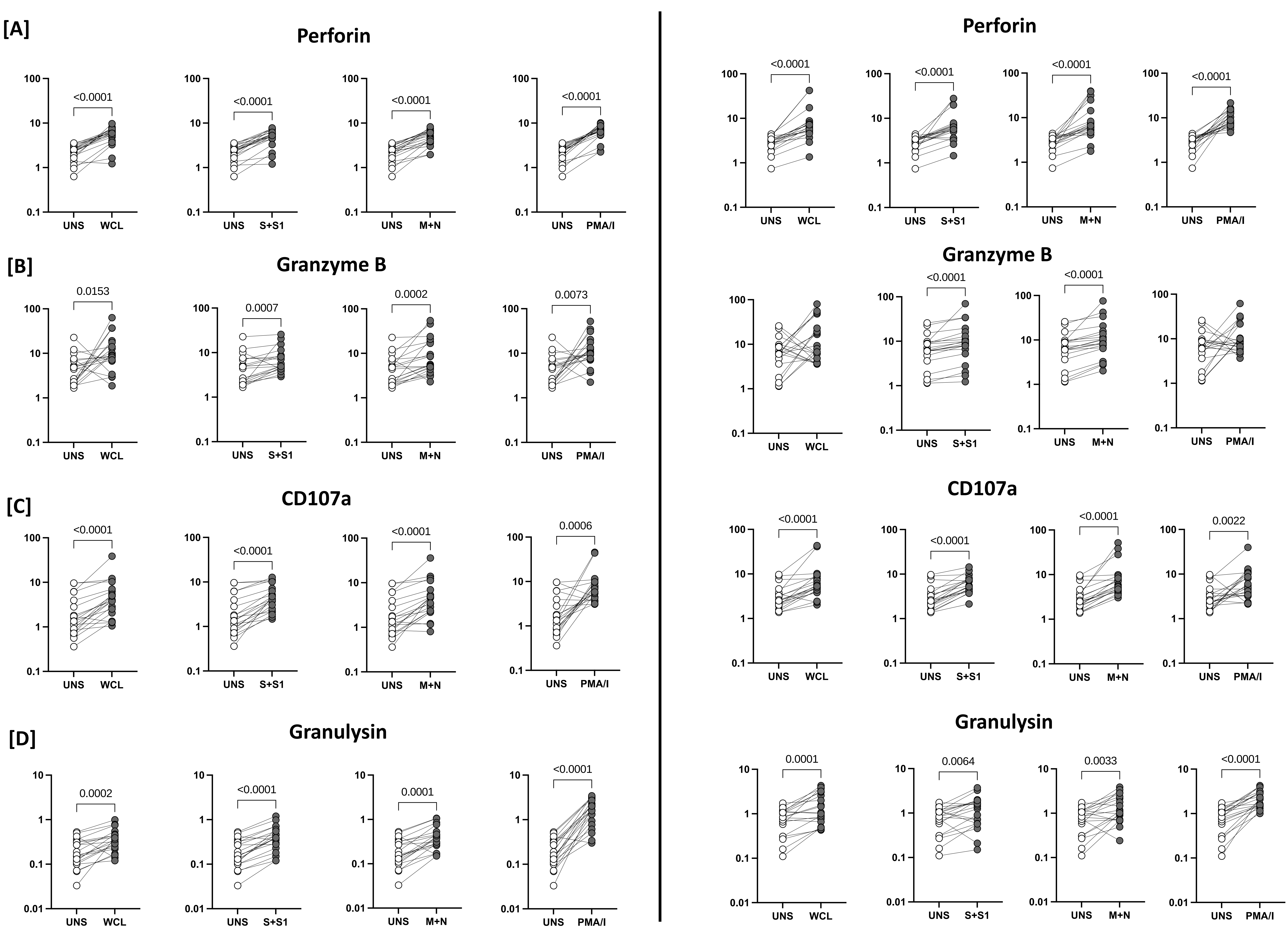

Figure S3: The frequencies of cytotoxic markers were estimated in ECV and LCV individuals upon no stimulation and SARS-CoV-2 antigen stimulation. Each circle represents a single individual and the bars represent the geometric mean values *P* values were calculated using the Wilcoxon matched pair test.
